# Managing daytime sleepiness with the help of sleepcoaching, a non-pharmacological treatment of non-restorative sleep

**DOI:** 10.1007/s11325-019-01995-0

**Published:** 2019-12-18

**Authors:** Brigitte Holzinger, Katharina Levec, Melissa-Marie Munzinger, Lucille Mayer, Gerhard Klösch

**Affiliations:** 1 Institute for Consciousness and Dream Research, Canongasse 13/, 1A-1180 Vienna, Austria; 2 Medical University, Vienna, Austria; 3 Department of Neurology, Medical University of Vienna, Vienna, Austria

**Keywords:** Daytime sleepiness, Holzinger and Kloesch ™, Sleep disorders, Sleep quality

## Abstract

**Purpose:**

To measure the effect of a 2-day sleepcoaching seminar on daytime sleepiness and sleep-related variables of shift workers employed in an Austrian railway company (ÖBB: Österreichische Bundesbahnen).

**Method:**

Participants filled in pre- and post-intervention questionnaires, containing items of the PSQI and the ESS, questions about chronotype, personality factors and possible burnout risk factors. About 30 shift workers, working in shifts for more than 300 months on average (28 male; mean age = 24 ± 45.90, age range 24–56 years) voluntarily took part in the investigation twice. Sleep coaching by Holzinger and Kloesch™ (SC) is a new holistic approach for non-pharmacological treatment of non-restorative sleep and is based on Gestalt therapy. It includes psychotherapeutic aspects, which enable clients to improve their sleep quality by developing one’s own coping strategies which can be implemented in daily routine. Dream work and relaxation techniques are also part of the programme.

**Results:**

The 2-day SC seminar was beneficial by focusing on the sleep problems related to shift work. A significant improvement of the global PSQI score and the PSQI variables subjective sleep quality, diurnal fatigue, and sleep latency was achieved, with a medium effect size. However, the programme did not result in the reduction of daytime sleepiness (ESS). Six more variables did not change significantly.

**Conclusion:**

While some sleep problems related to shift work were successfully addressed by SC, daytime sleepiness (ESS) could not be reduced contrary to our expectations. More research with a greater sample and a longitudinal design is needed to examine the long-term effects of SC.

## Introduction

A society accustomed to a permanent availability of communication and consumption requires a 24/7 service in various sectors. Working in (changing) shifts affects several aspects of life and is associated with increased rates of shift work sleep disorder. Two of the core symptoms are excessive daytime sleepiness and reduced alertness, which in turn interfere with the ability to engage in social activities [1]. Irregular work, disrupted sleep patterns, and desynchronised biological rhythms lead to reduced cognitive performance and an increased risk of accidents [2]. Therefore, the need for prevention and coping strategies for managing fatigue, daytime sleepiness, and decreased alertness is big. Klösch et al. [3] reported that evening chronotypes working in shifts had significantly lower subjective sleep quality and longer sleep latencies than morning chronotypes working in shifts. The following study investigates whether sleep coaching (SC), a non-pharmacological treatment of non-restorative sleep by Holzinger and Klösch [4], mediated in a 2-day seminar, improves quality of sleep and daytime functioning of shift workers.

## Methods

### Participants

This interventional prospective study included 30 shift workers (of whom 28 men; mean age = 24 ± 45.90, age range 24–56 years) of various occupations, who had worked in shifts continuously for 300 ± 126.54 months in average.

### Questionnaires

First, participants were asked to fill in demographic information.

They were then asked about their shift schedules and if their working schedule affected them *physically*, *psychologically*, *socially* or *mentally*. More than one answer was possible.

The participants then chose their chronotype of the three categories “morning”, “evening” or “in between”. Then they were asked about perfectionism and burnout, the questions about perfectionism being “Do others think you are a perfectionist?” and “Do you think you are a perfectionist?”. The burnout scale included the two questions “Do you often feel lack of energy?” and “Do you sometimes feel confused, as if not quite yourself?”. This could be seen critically, since burnout was assessed by only two questions, and its validation did not include already established measurements.

In addition, participants were asked if they suffered from sleep problems and if these problems affected their quality of life. Response categories to these six items ranged on a 4-point-scale from “not at all true” (0 points), “partly true” (1 point) to “mostly true” (2 points) and “completely true” (3 points). An average score was then calculated which led to a maximum score of three points in each category. This was followed by a 40-item self-rating questionnaire consisting of BFI-10 [5], PSQI, and ESS.

#### Pittsburgh sleep quality index (PSQI)

The PSQI (German version) [6, 7] assesses sleep quality. Based on seven components, *subjective sleep quality*, *sleep latency*, *sleep duration*, *habitual sleep efficiency*, *sleep disturbances*, *use of sleeping medication* and *daytime dysfunction,* a global score between 0 and 21 is calculated (21 = poorest sleep quality and 0 = no sleep problems), scores higher than 5 indicate poor sleep quality [6].

#### Epworth sleepiness scale (ESS)

The ESS measures daytime sleepiness. Participants rate the likelihood to doze off in everyday situations on a scale from 0 (*“would never doze”*) to 3 (*“high chance to doze”*) to gain a global score between 0 and 24 (24 = highest daytime sleepiness) [8]. Scores > 10 indicate critical daytime sleepiness.

### Sleepcoaching

Holzinger and Klösch [4] created the alternative concept of *sleepcoaching* by combining approved non-pharmacological strategies*.* The intention was to create an efficient treatment for sleep issues, using the most important aspects of cognitive and behavioural approaches and combining them with Gestalt Therapy (GT) ideas. SC consists of the four components *sleep education and sleep hygiene*, *cognitive behavioural therapy* (CBT), *hypnosis and relaxation* and *dream work*, all based on essential aspects of GT.

GT addresses biased perceptions and reveals suppressed emotions using role-playing games, feedback and sharing. Furthermore, it detects the source behind sleep problems by focusing on the “gestalt” of it, which is framed by maladaptive daytime behaviours.

Sleep education, sleep hygiene and CBT-techniques are included into SC to identify incorrect sleep attitudes and to correct maladaptive behaviour, providing strategies like stimulus control, sleep restriction and chronotherapy. Self-hypnosis, various relaxation techniques, autogenic training and meditation are part of the hypnosis component of SC [4, 9–11].

One part of the SC-approach is dream work, which provides coping strategies for nightmares and enhances positive impacts of lucid dreaming and working through dreams as an essential part of the sleep process [4]. This new holistic approach, provided by the Institute for Consciousness and Dream Research (ICDR), is an essential part of seminars and teaching courses, which are offered to private companies and patient groups. SC is also a postgraduate training course at the Medical University of Vienna.

### Elicitation

The intervention programme was offered to all employees of ÖBB, participation and completion of the survey were voluntary. The questionnaire was available online (www.*sleepcoaching.org**)* 14 days before participating in the SC*-*seminars, 6 months after the seminar participants could voluntarily fill it in again. In total, 184 shift workers completed the online survey in the time frame from 24.09.2015 to 04.06.2017, of whom 30 shift workers completed the survey before *and* after the seminar. Non-shift workers who took part in the programme were not included in this study.

### Statistical analysis

Data resulting from PSQI, ESS and additional variables of the questionnaire was extensively explored comparing the group results (*N* = 30) before and after the 2-day seminar to see if there were significant improvements. For this purpose, Wilcoxon tests were conducted. Two-tailed distributions were used. Effect sizes (Pearson r) were calculated. Results were presented as medians with interquartile ranges (IQR). Total scores in daytime sleepiness (ESS) were compared to see how many participants exceeded pre-defined cut-off scores. Chi-square tests were performed to see if distributions of critical scores in ESS differed significantly for the two groups. Participants were asked about the type of impact shift work had on their lives, and Wilcoxon tests were done to compare the results for the two points in time. For statistical analysis, the threshold for the rejection of the null hypothesis has been set to .05 but after Bonferroni correction for multiple comparisons was applied, the significant *p* value was reduced to 0.0038. The data used in the statistical analysis was collected in the time frame of 24.09.2015 to 04.06.2017. All statistical analyses were performed using SPSS-24 (Statistical Package for the Social Science).

## Results

Results provided in Table 1 show significant improvement of the global PSQI-score, *subjective sleep quality*, and a highly significant reduction of *diurnal fatigue* and *sleep latency* 6 months after the seminar. A medium treatment effect was found for all those variables. Changes in the variables *sleep disorders*, *consumption of soporifics*, *sleep duration*, *sleep efficiency, daytime sleepiness, burnout* and *impact of sleep issues on life quality* were nonsignificant.

**Table 1:** Descriptive statistics of the scores in PSQI, ESS, burnout, sleep issues, life quality and impact on life of shift workers before and 6 months after the SC seminar

	Before (*N* = 30)	After (*N* = 30)		Effect size
	Median (IQR)	Median (IQR)	*p* value	r**
PSQI				
Global score	7.0 (4.25)	4.5*(4.25)	0.002	0.383
Subjective sleep quality	1.0 (1.00)	1.0* (0.00)	0.002	0.414
Sleep disorders	1.0 (0.25)	1.0 (0.00)	0.289	0.183
Consumption of soporifics	0.0 (0.00)	0.0 (0.00)	1.000	0.129
Diurnal fatigue	1.0 (1.00)	1.0* (1.00)	0.002	0.414
Sleep duration	0.0 (1.00)	0.0 (1.00)	1.000	0.000
Sleep latency	2.0 (1.25)	1.0* (1.00)	0.002	0.402
Sleep efficiency	0.0 (1.25)	0.0 (1.00)	0.181	0.411
ESS				
Daytime sleepiness	9.0 (5.25)	6.0 (7.00)	0.004	0.365
Additional variables				
Burnout	1.0 (1.00)	1.7 (1.00)	0.427	0.188
Impact on life quality	2.0 (2.0)	1.0 (1.25)	0.005	0.114

*PSQI* Pittsburg sleep quality index

*ESS* Epsworth sleepiness scale

*significant at the *p* < 0.0038 level, according to Bonferroni-correction

**Cohen suggested that r = 0.1 be considered a small effect size, 0.3 a medium effect size and 0.5 a large effect size

In case of PSQI-score, the cut-off point (> 5) is still exceeded, but an improvement has been registered. ESS-mean-score does not exceed the cut-off point (> 10) of critical daytime sleepiness.

**Fig. 1:**
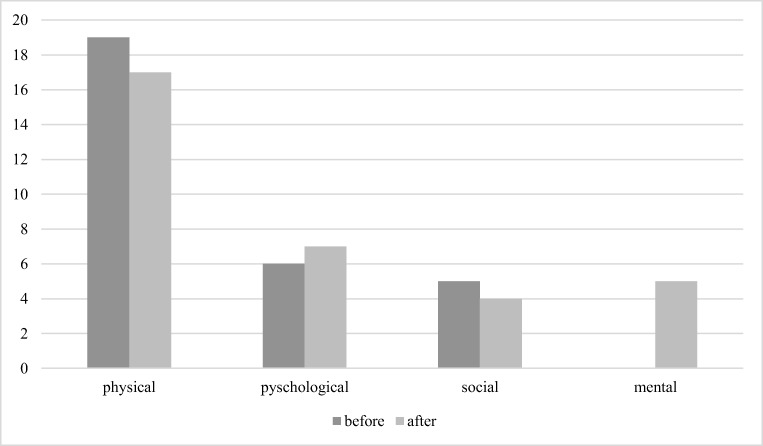
Distribution of complaints resulting from working schedules as reported by shift workers before and 6 months after the seminar. Note that the main impairment was of physical nature. Statistically significant differences were only observed for mental complaints

Regarding chronotypes (*N* = 30), 3 (10%) defined themselves as persons who feel active in the morning, 14 (46%) quoted to be active in the evening, 12 (40%) reported no preference in evening or morning time and one answer was missing. Perfectionism scores did not differ significantly for the two survey points [t(29) = 1.35, *p* = 0.187]. Impairments were mostly of physical nature (before: 63.3%, after: 56.7%), followed by psychological (before: 0.2%, after: 0.23%), social (before: 0.17%, after: 0.13%) and mental derogations (before: 0%, after: 0.17%) (see Fig. 1). The number of participants reporting physical impacts [Z(*N* = 30) = 0.82, *p* = .41], psychological impacts [Z(*N* = 30) = 0.45, *p* = .66] and social impacts [Z(N = 30) = 0.45, p = .66] did not change significantly. However, mental impacts were only reported after the seminar, and therefore the number of participants reporting them changed significantly [Z(*N* = 30) = 2.24, *p* = .025]. This might be due to an insufficient understanding of the term “mental impacts” before the seminar.

Between before and after the seminar, no significant difference in the distribution of ESS values above and below 10 (χ^2^ = 0.884, df = 1, *p* = 0.347) was found.

## Discussion

The present interventional study evaluates the effectiveness of a SC seminar on sleep problems and diurnal fatigue of shift workers at the Austrian Railways Company ÖBB. The 2-day SC-seminar addressing shift workers’ specific problems provides help by encouraging participants to be their own coach in disseminating information and skills. As a result, a significant improvement in the global PSQI-score, subjective sleep quality, diurnal fatigue and sleep latency was achieved. Daytime sleepiness (ESS), however, could not be improved due to the programme, contrary to our expectations. The same applies to the remaining variables sleep disorders, consumption of soporifics, sleep efficiency, sleep issues, sleep duration as well as burnout risk. This should be kept in mind for future adaptations of the programme. A longer, more in-depth treatment might be necessary to cure those issues that could not be improved in the 2-day seminar. Mental derogations as a result of shift work were only reported after the seminar, which might be due to the fact that the term was not clear for all participants at the first measurement. However, this should be explored further, since a higher number of mental complaints could also be a result of raising awareness during the seminar and then offering no further possibility to handle those complaints.

However, a number of issues seem to be addressed successfully. Sleep education and sleep hygiene help implementing strategies into daily pre-sleep routines and raise awareness of the risks of developing chronic sleep disturbances. Additionally, techniques of self-hypnosis and relaxation are explained to improve sleep onset latency and reduce wake time during sleep [12]. SC-methods were combined with cognitive behavioural techniques [13, 14]. Cognitive behavioural therapy (CBT) is proven to be successful in shortening sleep latency, improving sleep duration and sleep quality [15, 16]. In addition, GT empowers to locate the origin of their sleep problem and offers additional advices and techniques [17, 18].

The subcategories of the PSQI allowed further insight by addressing the social impacts of shift work. Sleep education and selected CBT techniques may have beneficial effects on sleep duration and quality [19]. In comparison to this approach, SC provides a more profound basis for treating sleep and wake behaviour to guarantee a more comprehensive understanding of the underpinnings that consolidate sleep complaints.

Furthermore, the influence of one’s chronotype was addressed in the SC-seminar, since this may influence the tolerability of shift work [20]. There is some evidence that evening types have more problems in getting back to work during daytime hours than morning types, while morning types showed higher sleep disturbances and social jetlag when working nights [21]. Hence, SC provides strategies for getting along better with time preferences for being active and sleeping habits.

Results show that the sole application of sleep hygiene is not useful in overcoming sleep issues [4] and self-hypnosis and relaxation techniques are no specific therapies by themselves [22]. They are meant to be taught and applied by clients on their own at home when needed [4]. Our findings showed an improvement in sleep latency, which can partly be associated with the usage of hypnosis, since studies revealed significant effects [10, 11, 13, 23].

During the seminar, all participants received information and instructions for relaxation techniques and rules for sleep education. Consequently, the application at home can be assured, enabling shift workers to integrate the techniques into their irregular work and life rhythms. Therefore, it seems that SC is a reasonably effective treatment option for sleep disorders in those shift workers who completed the programme.

Generalization of the presented results is limited, since participation in the seminar and completion of the survey were voluntary, and only a small number of participants filled in the questionnaire twice. Interest and motivation to take actions against the negative effects of shift work might have been bigger in participants taking part in the programme and even bigger in those completing the survey. This type of survey makes it difficult to evaluate if less motivated shift workers would also benefit from the programme. Non-shift workers were not included in this study, and those who were included were already in shifts for more than 300 months on average, 28 of 30 subjects being men. Further investigation with a larger sample and analysis of long-term effects is necessary. Moreover, it should be taken into account that only self-reported data was used for anamneses of and no other sources of information were used. Researchers might be biased towards the programme and its beneficial effects since it was developed by two of them; nevertheless, statistical analysis has been conducted conscientiously to avoid biased results. Chronotypes were only assessed by one question, in which participants chose themselves whether they are a morning type, evening type or in between. The Morningness-Eveningness Questionnaire by Horne and Östberg [24] could be included in future investigations. The same applies to burnout, which was only assessed by two questions and could instead be measured with validated instruments like the Maslach Burnout Inventory [25]. A significant reduction of daytime sleepiness (ESS) could not be achieved here and should be investigated with a larger sample in the future.

Nevertheless, sleepcoaching has been shown to raise awareness of existing sleep problems and was beneficial regarding some of the issues addressed in the programme. It should therefore be further investigated and enhanced in the future to allow for a more comprehensive cure of relevant sleep issues in the population.
